# CRISPR/Cas9 and *piggyBac* Transposon-Based Conversion of a Pathogenic Biallelic *TBCD* Variant in a Patient-Derived iPSC Line Allows Correction of PEBAT-Related Endophenotypes

**DOI:** 10.3390/ijms24097988

**Published:** 2023-04-28

**Authors:** Valentina Muto, Federica Benigni, Valentina Magliocca, Rossella Borghi, Elisabetta Flex, Valentina Pallottini, Alessandro Rosa, Claudia Compagnucci, Marco Tartaglia

**Affiliations:** 1 Molecular Genetics and Functional Genomics, Ospedale Pediatrico Bambino Gesù, IRCCS, 00146 Rome, Italy; federica.benigni@opbg.net (F.B.); valentina.magliocca@opbg.net (V.M.); rossella.borghi@opbg.net (R.B.); claudia.compagnucci@opbg.net (C.C.); marco.tartaglia@opbg.net (M.T.); 2 Department of Science, University Roma Tre, 00146 Rome, Italy; valentina.pallottini@uniroma3.it; 3 Department of Oncology and Molecular Medicine, Istituto Superiore di Sanità, 00161 Rome, Italy; elisabeta.flex@iss.it; 4 Neuroendocrinology Metabolism and Neuropharmacology Unit, IRCSS Fondazione Santa Lucia, 00143 Rome, Italy; 5 Department of Biology and Biotechnologies “Charles Darwin”, Sapienza University of Rome, 00185 Rome, Italy; alessandro.rosa@uniroma1.it; 6 Center for Life Nano- & Neuro-Science, Fondazione Istituto Italiano di Tecnologia (IIT), 00161 Rome, Italy

**Keywords:** iPSCs, CRISPR/Cas9 technology, gene editing, isogenic controls, neurodevelopmental disorder, TBCD, PEBAT

## Abstract

Induced pluripotent stem cells (iPSCs) have been established as a reliable in vitro disease model system and represent a particularly informative tool when animal models are not available or do not recapitulate the human pathophenotype. The recognized limit in using this technology is linked to some degree of variability in the behavior of the individual patient-derived clones. The development of CRISPR/Cas9-based gene editing solves this drawback by obtaining isogenic iPSCs in which the genetic lesion is corrected, allowing a straightforward comparison with the parental patient-derived iPSC lines. Here, we report the generation of a footprint-free isogenic cell line of patient-derived *TBCD*-mutated iPSCs edited using the CRISPR/Cas9 and *piggyBac* technologies. The corrected iPSC line had no genetic footprint after the removal of the selection cassette and maintained its “stemness”. The correction of the disease-causing *TBCD* missense substitution restored proper protein levels of the chaperone and mitotic spindle organization, as well as reduced cellular death, which were used as read-outs of the *TBCD* KO-related endophenotype. The generated line represents an informative in vitro model to understand the impact of pathogenic *TBCD* mutations on nervous system development and physiology.

## 1. Introduction

Induced pluripotent stem cells (iPSCs) are stem cells reprogrammed from adult somatic cells with different embryonal origins (endoderm, ectoderm, or mesoderm) that can be differentiated in vitro into functional multilineage mature cells [1]. IPSCs meet the defining criteria of pluripotent stem cells (i.e., self-renewal ability, and in vitro differentiation potential). They can be differentiated into any cell lineage in an ontogeny-recapitulating manner [1], allowing the opportunity to generate informative in vitro disease models. IPSCs have been successfully used to explore the pathogenetic mechanisms and pathophysiology of a wide range of neurological diseases [2]. Notwithstanding this unique potential, there are issues that need to be addressed for their reliable use. A major concern is related to the variability in the differentiation potential of different clones that are obtained from the same parental cell line [3]. This limitation is intrinsic to this model system and may result in an inappropriate interpretation of the disease endophenotype. Variability is regularly observed also in control iPSCs derived from healthy individuals, which complicates the understanding of the findings when comparing iPSC-derived differentiated cells obtained from patients and healthy subjects. The generation of isogenic iPSC lines (i.e., cells with a genome identical to the parental ones in which the disease-causing mutation(s) has (have) been corrected) allows this drawback to be overcome [4]. For this reason, an increasing number of studies aimed at understanding the mechanism of disease use patient-derived iPSCs and their isogenic controls obtained by gene-editing techniques [5,6,7,8,9,10,11].

The clustered regularly interspaced short palindromic repeats (CRISPR)/Cas9 system is a widely used gene-editing tool that is considered fast, easy, and cheap. It requires the generation of the guide RNA (gRNA) containing a 20-nucleotide homologous sequence followed by a trinucleotide (NGG) protospacer adjacent motif (PAM) in the target, and expression of a CRISPR-associated (Cas) endonuclease [12]. Despite this acknowledgment, the application of the CRISPR/Cas9 technology to iPSC modeling still does not represent an easy endeavor, as homologous recombination (HR), which is its rate-determining step, occurs with extremely low efficiency in iPSCs [13], and recovery of correctly targeted clones without positive selection is labor-intensive and with unpredictable efficiency. Moreover, genome editing in iPSCs is also difficult due to their tendency to encounter programmed cell death when they are cultured as single cells, requiring the use of reporter systems or selectable markers to facilitate the identification of the rare recombination events. After selection, the reporter or selection marker needs to be removed, and tools (e.g., the *piggyBac* (PB) transposon system) have been designed to accomplish this goal in a footprint-free manner [14,15,16,17,18].

We previously reported that biallelic hypomorphic/loss-of-function variants in *TBCD*, the gene encoding the tubulin folding cofactor D, one of the five co-chaperones required for assembly and disassembly of α/β-tubulin heterodimer, perturb microtubule dynamics and cause a recessive neurodevelopmental/neurodegenerative disorder (PEBAT; MIM 617193) [19]. The clinical phenotype is relatively homogeneous, even though variability in the onset and progression is observed. The presence of microcephaly in a large proportion of affected individuals, the progressive nature of the disease, and the occurrence of cortical atrophy and hypomyelination followed by cerebellar atrophy indicate that this disorder is characterized by both neurodevelopmental and neurodegenerative features, in which the less severe phenotypes are characterized by early-onset neurodegeneration [19,20,21,22,23,24,25]. In patient-derived fibroblasts, defective TBCD function affects the assembly and disassembly of αβ-tubulin polymers, resulting in a shift toward a more rapidly growing and more stable microtubule population [19]. At the centrosome, TBCD is required for the initiation of microtubule growth and organization of the mitotic spindle [26,27]. Consistently, we documented an altered spindle structure in patients’ fibroblast lines, with disorganized, tangle-shaped mitotic microtubules, and markedly reduced aster formation [19].

Here, we combined the CRISPR/Cas9 and *piggyBac* transposon technologies to generate a footprint-free knock-in isogenic iPSC line from a parental iPSC line carrying a homozygous inactivating variant in *TBCD* and validate the conversion event by assessing features of the PEBAT-associated endophenotype.

## 2. Results

### 2.1. CRISPR/Cas9-Induced HR in iPSCs

**Figure 1:**
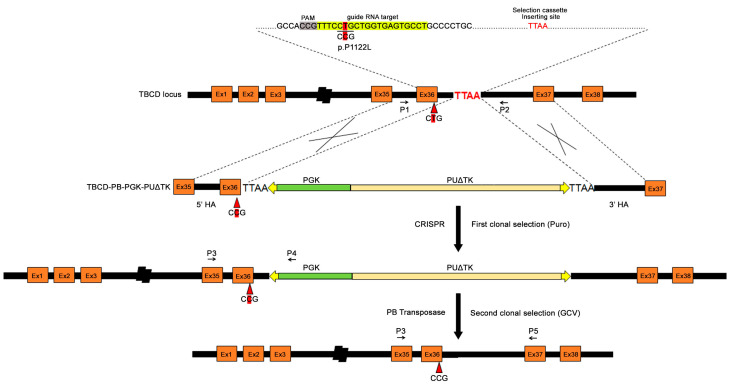
Schematic representation of the strategy for generating template DNA for HR-based gene editing. The position of the variant at codon 1122 (CTG to CCG), guide RNA target/PAM, and selection cassette insertion site (TTAA) are highlighted at the *TBCD* locus (top). The c.3365C>T (p.P1122L) variant is located on exon 36. The targeting donor plasmid includes PGK, phosphoglycerate kinase 1 promoter; PUΔTK, a fusion between PuroR and DeltaTK (truncated version of HSV type 1 thymidine kinase) and two homologous arms (HA). Yellow triangles represent enhanced *piggyBac* (ePB) terminal repeats. Short arrows in black indicate the positions of primers used for PCR amplification of the fragments sequenced.

**Figure 2:**
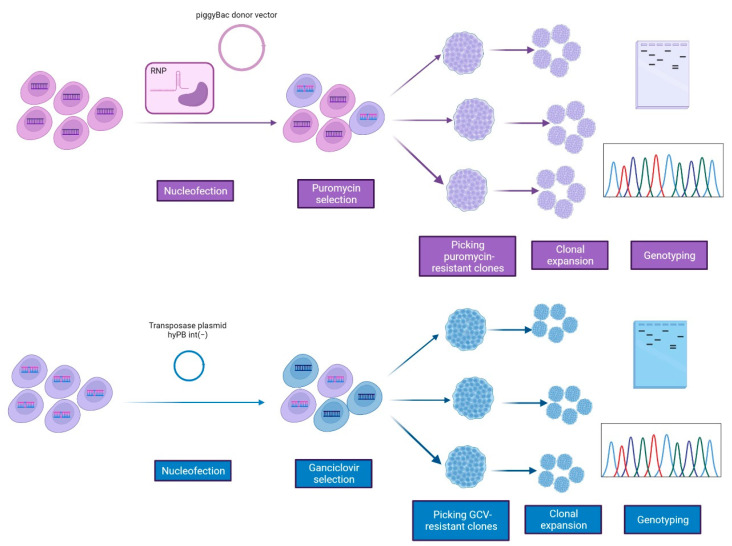
Cartoon depicting the workflow used for the generation of an isogenic cell line of the *TBCD*-mutated iPSC line (p.Pro1122Leu) using the combination of the CRISPR/Cas9 and *piggyBac* technologies.

**Figure 3:**
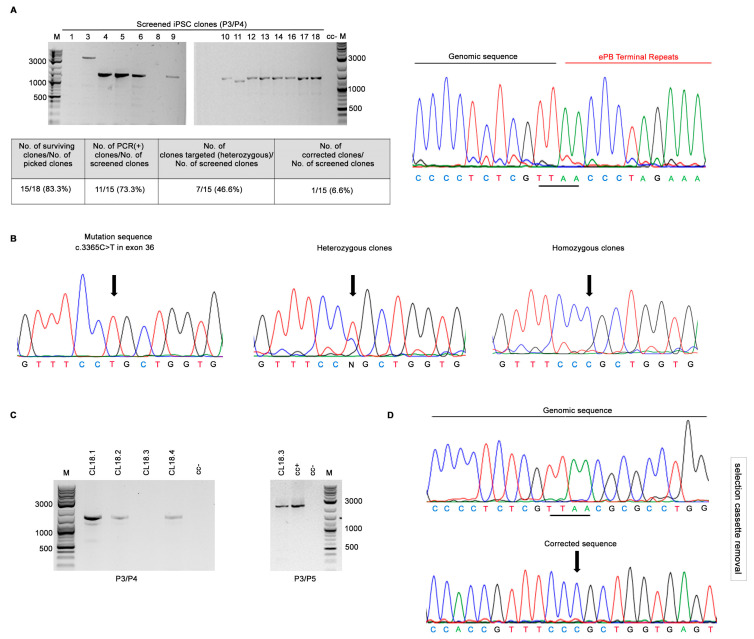
Gene correction of the pathogenic *TBCD* missense change (c.3365C>T, p.Pro1122Leu) in patient-derived iPSCs using the CRISPR/Cas9 system and *piggyBac* transposon. (**A**) After puromycin exposure, sequencing of the PCR product (primers P3/P4, ~1400 bp) shows the junction between the PB terminal repeats and genomic sequences before transposon removal. (**B**) Direct sequence analysis was performed as a screening test, resulting in 7 clones in which HR occurred in only one allele (heterozygous clones) and 1 clone in which HR occurred in both alleles (homozygous clone). (**C**) After selection cassette removal, PCR analysis using primers P3/P4 detected no fragments of the selection cassette in clone 18.3, while positive amplification with P3/P5 confirmed PB removal (~2000 bp). (**D**) Direct sequence analysis shows correction of the gene mutation and no insertions/deletions around the TTAA site in which the selection cassette was pre-inserted.

We used the CRISPR/Cas9 nuclease system together with the *piggyBac* transposase approach to correct the homozygous pathogenic *TBCD* variant, c.3365C>T (p.Pro1122Leu), in a previously generated and characterized iPSC line (Compagnucci C. and Tartaglia M., unpublished data). To ensure proper targeting in the *TBCD* gene, we selected one specific guide-RNA sequence (gRNA1) using Benchling’s CRISPR tool and the Wellcome Trust Sanger Institute Genome Editing database. To correct the *TBCD* c.3365C>T change in the parental patient-derived iPSCs (TBCDmut-iPSCs), we constructed a targeting donor plasmid using two ~700 bp-long segments (including the mutation site) upstream and downstream of the TTAA site, as homologous arms (Figure 1). The TBCD-PB-PGK-PUΔTK donor vector was nucleofected together with the gRNA1 and Cas9 nuclease in *TBCD*-mutated iPSCs. Following incubation with puromycin for positive selection, 18 drug-resistant colonies were obtained (Figure 2). Among these, 15 colonies were expanded and tested for homologous recombination by PCR amplification using primers P3 (annealing in a region of the *TBCD* gene upstream of the targeting construct) and P4 (annealing in the *piggyBac* cassette). The presence of the integrated selection cassette in the correct genomic position was verified in 11 iPSC colonies (73.3%) (Figure 3A). Sanger sequencing was then performed to select the clones in which HR-mediated gene correction occurred, resulting in seven clones in which the gene correction occurred in one allele (46.6%) (7/15) and one clone (clone 18) in which the correction occurred in both alleles (6.6%) (1/15) (Figure 3B).

### 2.2. Transposon Excision in the Corrected iPSCs

Since the parental iPSC line carries a homozygous inactivating variant in *TBCD*, we performed the transposon excision only in the homozygous clone. Specifically, for the selection cassette removal, clone 18 was transiently transfected with a modified PB transposase [hyPB int(−)], unable to re-integrate a sequence flanked by the PB terminal repeats [28]. After negative selection using ganciclovir, we obtained 4 resistant clones, which were screened by PCR using a pair of primers, P3/P4. Only one clone (clone 18.3) was not amplified by these primers and was therefore considered free of the PB transposon (Figure 3C). Subsequently, primers P3 and P5, which mapped in a region of the *TBCD* gene located outside of the targeting construct (~2000 bp) was used to confirm the presence of the edited *TBCD* genomic region by PCR (Figure 3C). By DNA sequencing, we validated the introduction of the conversion event and the absence of other sequence changes (Figure 3D). The excision efficiency was 25% (1/4 clones examined).

### 2.3. No Occurrence of Off-Target Event by CRISPR/Cas9 Gene Editing

**Figure 4:**
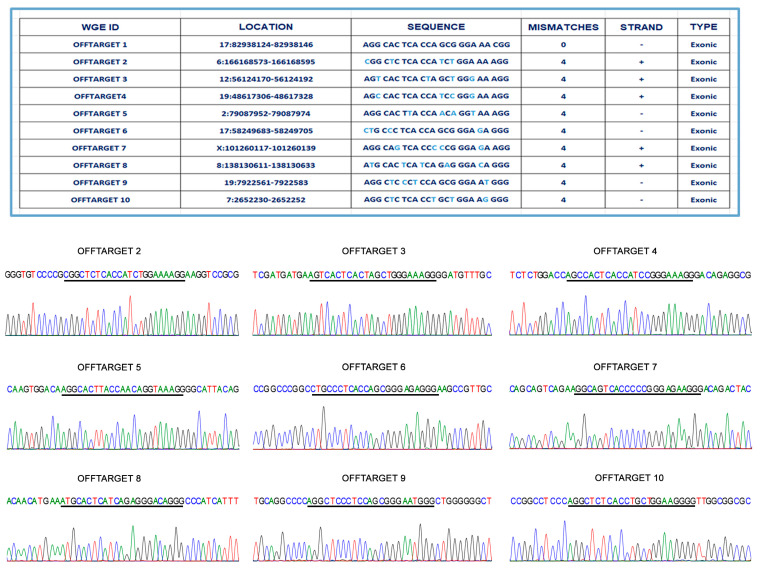
Off-target analysis of designed CRISPR/Cas9 system. By direct sequencing analysis, no off-target events were detected at nine candidate sites within exonic regions of the genome.

The targeted gene correction is expected to have a low impact on the whole-genome mutational load in human ES and iPSC lines [29,30]. To confirm this assumption, we excluded off-target events in the edited iPSC line by checking the occurrence of the top nine potential off-target sites that had been predicted by the Wellcome Trust Sanger Institute Genome Editing database (https://wge.stemcell.sanger.ac.uk//, accessed on 19 October 2021). For each site, we designed pairs of primers that covered the predicted indel, and Sanger sequencing analysis demonstrated the absence of any off-target event (Figure 4).

### 2.4. Isogenic iPSCs Retain Their Pluripotent Behavior and Genomic Integrity

**Figure 5:**
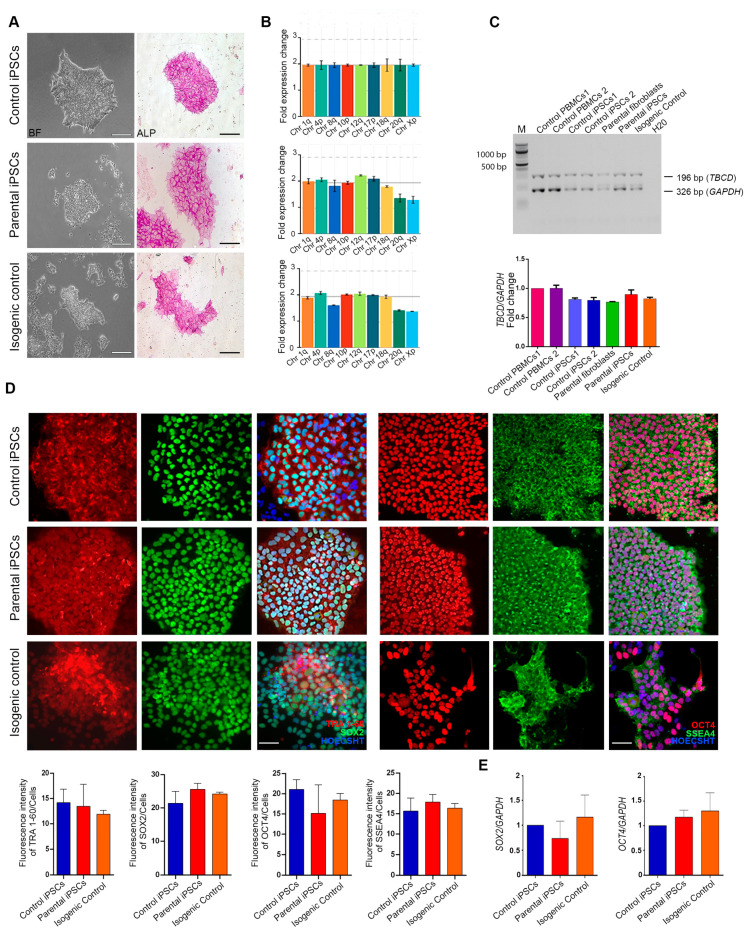
Pluripotency maintenance of the corrected isogenic iPSCs. (**A**) Brightfield images showing representative embryonic stem cell-like colony morphology for control iPSCs, patient-derived *TBCD*-mutated iPSCs, and isogenic iPSCs. Alkaline phosphatase assay was performed on parental iPSCs, isogenic lines, and control iPSCs. Scale bar = 100 μm. (**B**) Real-time PCR assay showing that the three iPSC lines do not present statistically significant structural rearrangements. (**C**) Agarose gel (2%) electrophoresis of amplified genomic DNA products in several samples using multiplex PCR. The arrows indicate a band of 326 bp corresponding to the amplified *GAPDH* product and a band of 196 bp corresponding to the amplified *TBCD* product. Lane 1: molecular weight marker; Lane 2 and 3: genomic DNA from human peripheral blood mononuclear cells; Lane 4 and 5: genomic DNA from control iPSCs; Lane 6: genomic DNA from patient-derived fibroblasts; Lane 7: genomic DNA from parental iPSCs; Lane 8: genomic DNA from the generated isogenic control line. The bar graph below represents the ratio of densitometry values of the target region to the reference region (*TBCD/GAPDH* product ratio). Data are normalized to control and presented as the mean ± SEM, *n* = 2. Kruskal–Wallis followed by Dunn’s *post hoc* tests are used to assess statistical significance. (**D**) Immunofluorescence assays demonstrating positive immunostaining for the pluripotency markers SOX2, TRA 1-60, OCT4, and SSEA 4 in control iPSCs, parental iPSCs, and control isogenic line. Scale bar = 50 μm. The bar graphs show the signal quantification of pluripotency markers in relation to the total number of cells. Data are presented as mean ± SEM; three biological replicates are shown as *n* = 3, according to an ordinary one-way ANOVA parametric test. (**E**) The bar graph shows the maintenance of pluripotency of the gene-edited iPSC lines, as demonstrated by the expression of *SOX2* and *OCT4* genes. Data are normalized to control and presented as the mean ± SEM; three biological replicates are indicated as *n* = 3. A one-way ANOVA parametric test is used to assess statistical significance.

To confirm the pluripotency of the corrected isogenic iPSC line, we tested its positivity to alkaline phosphatase (Figure 5A) and expression of a panel of stem cell markers. Immunostaining of OCT4, SOX2, TRA-1-60, and SSEA4 (Figure 5D) and qPCR analysis of *OCT4* and *SOX2* (Figure 5E) indicated that the selected iPSC line retained pluripotency following gene editing. DNA integrity was verified by assessing the eight most common karyotype abnormalities reported in iPSCs by a genetic analysis assay (Figure 5B). A multiplex competitive PCR using *TBCD*- and *GAPDH*-specific primer pairs confirmed the occurrence of two corrected copies of the gene in the isogenic cell line (Figure 5C). The isogenic cells also preserved the capability to proliferate (Figure S1A) and differentiate into the three embryonic germ layers, as shown by the expression of NCAM (ectoderm), SOX17 (endoderm), and brachyury (mesoderm) (Figure S1B).

### 2.5. Correction of the Pathogenic TBCD Variant Restores the Level of the Protein

**Figure 6:**
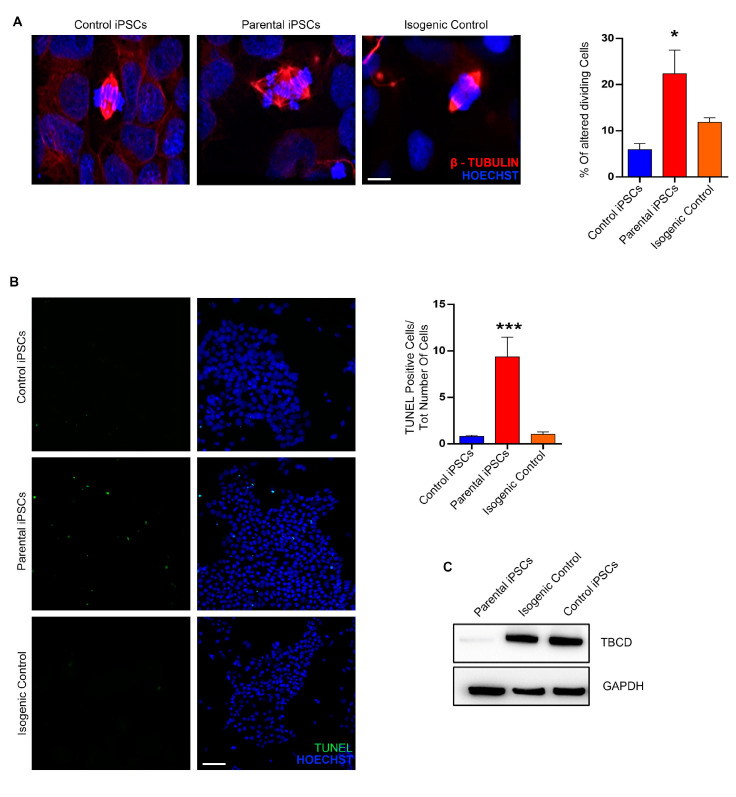
Rescue of TBCD protein levels and aberrant mitotic spindle structure following correction of the homozygous *TBCD* gene mutation in isogenic control lines. (**A**) Immunofluorescence analyses for β–tubulin showing a rescue of the mitotic spindle structures in the isogenic control line, in comparison with the impaired spindle microtubule organization in parental iPSCs. Scale bar = 50 μm. The bar graph represents the percentage of abnormal metaphases observed in dividing cells. Data are presented as mean ± SEM (normalized to control), *n* = 3. (**B**) TUNEL assay showed numerous parental iPSCs with fragmented DNA compared with control iPSCs; on the contrary, the number of apoptotic cells is visibly reduced in the isogenic control line. Scale bar = 25 μm. The graph represents the number of TUNEL-positive cells on the total number of cells. More than 3000 cells were analyzed for each sample. Data are presented as mean ± SEM (normalized to control), *n* = 3. Ordinary one-way ANOVA parametric test (**A**,**B**) is used to assess statistical significance, * *p* < 0.05, *** *p* < 0.0005. (**C**) Western blot analysis performed on control iPSCs, parental iPSCs, and isogenic control line indicated that the reduced levels of mutated TBCD protein (133 kDa) were restored in the isogenic control line.

Previous work showed that TBCD levels are significantly decreased in fibroblasts homozygous for the p.Pro1122Leu amino acid substitution compared to control cells, due to accelerated degradation of the mutated protein [19]. TBCD protein levels were assessed in the isogenic cell line, parental iPSC line carrying the homozygous disease-causing mutation, and control iPSCs by Western blot analysis, indicating that the correction of the pathogenic variant was associated with restored levels of the TBCD protein (Figure 6C).

### 2.6. Correction of the Pathogenic Homozygous TBCD Variant Restores the Alteration of Mitotic Spindle Structure Associated with Loss of TBCD Function

TBCD localizes at the centrosome and midbody, where it participates in centriologenesis, spindle organization, and cell abscission [26,27]. We previously demonstrated that patient-derived fibroblasts expressing biallelic pathogenic *TBCD* variants exhibit disorganized, tangle-shaped mitotic microtubules, and an altered spindle structure [19]. This endophenotype was confirmed in the parental patient-derived iPSC lines (Figure 6A). To investigate the rescue of this feature in the edited iPSC line, we performed confocal microscopy analysis using β-tubulin as a marker of the mitotic spindle. In contrast to what was observed in parental iPSCs, the altered spindle microtubule organization was rescued in isogenic iPSCs (Figure 6A). Moreover, the increased apoptotic rate of parental iPSCs associated with the altered spindle microtubule organization was reversed in the corrected iPSC line (Figure 6B). Overall, these findings provide evidence that correction of the homozygous pathogenic *TBCD* variant results in a rescue of pathological endophenotypes associated with PEBAT.

## 3. Discussion

The advent of iPSC technology has revolutionized the use of human in vitro models for neurodevelopmental/neurodegenerative disorders [31]. Indeed, iPSC-derived cells have been increasingly used for investigating molecular and cellular pathophysiological mechanisms underlying inherited diseases. In particular, iPSC modeling has successfully been employed to model neurologic disorders and diseases in which the pathophysiology is not recapitulated by animal models. Nevertheless, a drawback in using iPSCs as a model system is linked to the difficulty of properly ascribing the observed phenotype to the disease-causing mutation(s). This issue can be overcome by genome editing introducing the disease-associated mutation(s) of interest into control iPSCs or, alternatively, correcting the genetic lesion(s) in patient-derived iPSCs, in order to generate isogenic cell line pairs with identical genetic backgrounds, differing only by the presence/absence of the disease-causing variant of interest [32].

Precise genome editing in human iPSCs has historically been challenging; however, in the past decade, researchers have performed many studies to improve the efficiency of genome editing [33]. Among these, CRISPR/Cas9 technology represents the most powerful strategy, allowing the introduction or correction of specific mutations [34]. Hence, the combination of iPSC technology with CRISPR/Cas9 gene editing offers unprecedented opportunities to develop in vitro disease models. To name a few, CRISPR-Cas9 technology has successfully been applied to generate informative models for amyotrophic lateral sclerosis [35], Huntington’s disease [36], Duchenne muscular dystrophy [37], and inherited retinal degeneration [38]. Recently, the same technology in human progenitor cells (HSPCs) has been applied as a precise genome editing tool for treating beta-thalassemia and sickle cell disease [39]. Despite the great potential of iPSC-genome engineering using the CRISPR/Cas9 system, the laborious clonal selection remains a critical step. While this problem can be solved by the introduction of reporter systems or selectable markers, the safe removal of the cassette selection remains a critical issue. Given its ability to excise an exogenous DNA sequence completely from the genome in a footprint-free manner, the CRISPR/Cas9-associated *piggyBac* transposon system has become a valuable tool for targeted genetic manipulation [17,18,40,41,42].

TBCD is a microtubule manufacturing protein that in concert with four additional chaperones (TBCA, TBCB, TBCC, TBCE) and Arl2 is part of the molecular machinery required for the polymerization/depolymerization of MT and is thus essential to MT dynamics [43]. TBCD has been involved in centriole biogenesis and participates in the assembly of cilia and flagella, which are important to cell proliferation and differentiation during development [26,27,44]. Moreover, the roles of TBCD in MT dynamics appear crucial to the production of neuronal progeny, neuronal migration, and the development of synaptic connectivity between cortical postmitotic neurons, glial cells, and oligodendrocytes [45,46]. Variants in genes encoding tubulins and microtubule-associated proteins, which alter the microtubule function and dynamics, have been associated with human cortical malformations and neurodevelopmental disorders [47,48,49,50,51,52]. In particular, pathogenic mutations affecting *TBCD* have been shown to underlie PEBAT, an early-onset progressive encephalopathy characterized by brain atrophy. There is biochemical and functional evidence supporting the pathogenic effects of the *TBCD* variants on protein synthesis/stability/function, resulting in aberrant microtubule dynamics and altered mitotic spindle organization [19,22,23]. Despite the central importance of genes encoding tubulins and microtubule-associated proteins in MT dynamics, which play important roles in neurodegenerative and neuronal function maintenance, relatively few in vitro models of tubulin variants are available. ENU mutagenesis experiments on murine models have identified alleles in *Tuba1a* and *Tubb2b*, and a single mouse model for CFEOM (*Tubb3*) [53] and a Zebrafish model for PEBAT (*tbcd*) [21] have been generated.

Here, we generated an isogenic human model of iPSCs from a patient with PEBAT using CRISPR/Cas9 genome editing. We show that the generated isogenic iPSC line retains pluripotency and normal karyotype and it is capable of differentiating into the cells of the three embryonic layers. Importantly, the correction of the homozygous *TBCD* mutation restored proper TBCD protein levels, which are crucial for normal neuronal morphogenesis [21] and rescued the aberrant spindle morphology associated with defective TBCD function. These results further support the notion that defective TBCD function underlies the disease mechanism of this rare neurodevelopmental and neurodegenerative disorder and indicate a fundamental function for TBCD in the fine-tuning of the assembly and disassembly of the microtubule network.

The use of the presently generated in vitro model provides unique opportunities to explore the pathophysiological mechanisms underlying TBCD loss of function in the proper cellular context. This model is also expected to provide an experimental tool to identify and/or validate effective targeted therapeutic approaches directed to counterbalance the aberrant MT dynamics characterizing PEBAT.

## 4. Materials and Methods

### 4.1. gRNA Design and Donor Vector Construction for HR 

For CRISPR/Cas9-induced gene editing, gRNA was designed using two in silico software tools (Benchling’s CRISPR tool and Wellcome Trust Sanger Institute Genome Editing database (https://www.benchling.com/crispr and https://wge.stemcell.sanger.ac.uk//, accessed on 17 January 2018)) to target close to the c.3365C>T variant in *TBCD* exon 36. Once designed, gRNA was ordered as two-component CRISPR RNA (crRNA) and transactivating CRISPR RNA (tracrRNA) (IDT Corporation, Newark, NJ, USA) and then assembled at 95 °C for 5 min at room temperature.

To correct *TBCD*-mutated iPSCs, the donor plasmid was generated by amplifying a segment of ~1400 bp using the patient’s genomic DNA as a template, in which the causative variant is present (chr17:80,896,008). The genomic segment was subcloned into the pGEM-T Easy Vector (Promega, Madison, WI, USA), and the wild-type (WT) allele was introduced by site-directed mutagenesis using the QuikChange II Site-Directed Mutagenesis Kit (Agilent Technologies, Santa Clara, CA, USA). The selection cassette flanked by the enhanced *piggyBac* (ePB) terminal repeats and containing an independent promoter (PGK) driving the expression of the PUΔTK bifunctional protein [54], which confer resistance to puromycin and sensitivity to ganciclovir (GCV), was amplified using a targeting donor plasmid as previously described [55]. Subsequently, exploiting the restriction site for the HpaI enzyme (GTT^AAC), the donor vector was digested by HpaI. The drug-mediated selection cassette was flanked by the PB terminal repeats and inserted between two TTAA sites on each homologous arm (HA) (Figure 1). Primers for donor vector generation are summarized in Table S1.

### 4.2. Generation of Patient-Derived iPSCs

The studies were conducted in compliance with the Code of Ethics of the World Medical Association (Declaration of Helsinki), and with national legislation and institutional guidelines (local institutional ethical committee, Ref. 2357_OPBG_RC_2020, date of approval 19 February 2021). *TBCD*-mutated iPSCs were obtained from primary skin fibroblasts of an affected male individual (c.3365C>T, p.Pro1122Leu) with informed consent, and control iPSCs were purchased from System Biosciences. Cells were reprogrammed in house using non-integrating episomal technology as described in Borghi R. et al. (2021) [56].

### 4.3. Maintenance of Human iPSCs

The iPSC lines derived from the patient, those genetically corrected, and control iPSCs were all grown in feeder-free conditions using matrigel (Corning Inc., Corning, NY, USA) in mTeSR Plus (Stem Cell Technologies, Vancouver, BC, CA) and incubated at 37 °C, 5% CO_2_. The medium was changed every other day and the cells were split and transferred to new 6-well plates when they were 70–80% confluent.

### 4.4. CRISPR-Cas9 Gene Editing

To correct the pathogenic mutation, *TBCD*-mutated iPSCs were nucleofected using an IDT protocol. Briefly, crRNA (200 μM) and tracrRNA (200 μM) were assembled at 95 °C for 5 min to a final duplex concentration of 100 μM and then incubated with Cas9 nuclease (60 pmol) (IDT Corporation) for 20 min at room temperature. RNP complex and donor vector (2 μg) were then mixed with 200,000 single-cell *TBCD*-mutated iPSCs in P3 Primary Cell Nucleofector solution (Lonza, Morrisville, NC, USA). Samples were subsequently nucleofected using a 4D-Nucleofector System (Lonza). iPSCs were then seeded on a 6-well plate in mTeSR Plus medium added with 10 μM Y27632 (Sigma Aldrich, St. Louis, MO, USA). Two days after nucleofection, 0.5 mg/mL puromycin (Sigma Aldrich) was added for positive drug selection. After 14 days, visible colonies of iPSCs were mechanically isolated and expanded. PCR amplification and Sanger sequencing were performed to test for homologous recombination.

### 4.5. DNA Extraction, PCR, Multiplex PCR and Sanger Sequencing

Genomic DNA was extracted from 10,000 cells (1 well of a 96-well plate) using 100 μL genomic DNA extraction solution from a Quick Extract Kit (EPICENTRE Biotechnologies, Madison, WI, USA). PCR reactions were performed using KAPA2G Fast ReadyMix (KAPA Biosystems, Salt River, Cape Town, South Africa) and 0.4 μM forward and reverse primers for a total of 35 cycles. For multiplex PCR, the reaction was performed using 50 ng of DNA for 28 cycles. The amplification primers for *TBCD* and *GAPDH* were designed to simultaneously amplify target fragments from genomic DNA of 196 bp and 326 bp, respectively.

Sanger sequencing analysis was performed with an Applied Biosystems 3730xI DNA analyzer, using the ABI Prism BigDyeTM Terminator Cycle Sequencing Ready Reaction Kit ver.3.1. (Applied Biosystems, Waltham, MA, USA). Primers for PCR and sequencing are summarized in Table S1.

### 4.6. RNA Extraction and Real-Time qPCR

Total RNA was extracted from iPSCs using TRIzol reagent (ThermoFisher, Waltham, MA, USA) according to the manufacturer’s protocol. The reverse transcription reaction was performed with 1 µg of total RNA, and cDNA was generated by the SuperScript IV First-Strand Synthesis System (ThermoFisher) using random hexamers. RT-qPCR was performed using Fast SYBR Green Master Mix (Applied Biosystems) and a QuantStudio 7 Pro Real-Time PCR System (ThermoFisher) according to the manufacturer’s instructions. Primers for qPCR are summarized in Table S1. Relative changes in gene expression were calculated using the 2^−ΔΔCt^ method. Quantitative RT-PCRs were repeated in triplicate from at least two independent experiments. 

### 4.7. Removal of Selection Cassette from Correct TBCD-Mutated iPSCs

To remove the PB-PGK-PUΔTK selection cassette, cells were nucleofected with 5 μg of the hyPB int(−) piggyBac transposase [39]. After 7 days, 40 μM ganciclovir (Sigma Aldrich) was added to the medium for negative drug selection, and surviving cells were cultured until iPSC colonies appeared. The clones were then individually isolated for expansion and characterization.

### 4.8. Off-Target Analysis

We used the CRISPR/Cas9 target prediction tool “Wellcome Trust Sanger Institute Genome Editing database” (https://wge.stemcell.sanger.ac.uk//, accessed on 19 October 2021). The top 9 potential exonic off-target sites were analyzed. We chose off-target sites in exonic regions with 4 mismatches, as there were no off-target sites with fewer mismatches. For each site, we designed products that spanned ~500 bps of the predicted indel region and performed a Sanger sequencing analysis to confirm the absence of mutations.

### 4.9. Immunoblotting Assay

IPSCs were lysed in radio-immunoprecipitation assay (RIPA) buffer, pH 8.0, containing phosphatase and protease inhibitors (Sigma-Aldrich). Lysates were kept on ice for 30 min and centrifuged at 16,000× *g* for 20 min at 4 °C. Samples containing an equal amount of total proteins (20 μg) were resolved by 7.5% sodium dodecyl sulfate (SDS)-polyacrylamide gel (Biorad, Hercules, CA, USA). Proteins were transferred to nitrocellulose membrane using a dry transfer system (Biorad), and blots were blocked with 5% non-fat milk powder (Biorad) in Phosphate-buffered saline (PBS) containing 0.05% Tween-20 for 1 h at 4 °C and incubated with mouse monoclonal anti-TBCD (1:500, ThermoFisher), mouse monoclonal anti-GAPDH (1:1000, Santa Cruz, CA, USA) and anti-mouse HRP-conjugated secondary antibody (1:3000, ThermoFisher). Immunoreactive proteins were detected by an enhanced chemiluminescence (ECL) detection kit (ThermoFisher) according to the manufacturer’s instructions, and an Alliance Mini HD9 (Uvitec) was used for chemiluminescence detection.

### 4.10. Immunofluorescence Assay

IPSCs were seeded in 24-well cluster plates onto 12 mm cover glasses and fixed in 4% paraformaldehyde (Cell Signaling Technology, Danvers, MA, USA) for 10 min at room temperature. Cells were then blocked with 5% bovine serum albumin (BSA) (Roche, Basilea, Switzerland) followed by permeabilization with 0.1% Triton X-100 (Sigma Aldrich) for 1 h at room temperature. To assess the pluripotency, we used a Pluripotent Stem Cell 4-Marker Immunocytochemistry Kit (Invitrogen, Waltham, MA, USA), according to the company’s instructions. Primary antibodies were incubated for 3 h at room temperature and included anti-OCT4 (1:100, rabbit), anti-SSEA4 (1:250, mouse), anti-SOX2 (1:200, rat), and anti-TRA-1-60 (1:100, mouse). To analyze the mitotic spindle, we used anti-β-tubulin antibody (1:500, mouse). Secondary antibodies were added in blocking buffer for 1 h at room temperature. Nuclei were stained with 1:10,000 Hoechst 33342 (Invitrogen).

### 4.11. Alkaline Phosphatase Assay

IPSCs were plated on slides, washed with PBS, and fixed with 4% PFA for 10 min at room temperature. ALP staining was carried out using the Phosphatase Alkaline Kit (Sigma Aldrich). Cells were incubated for 30 min at room temperature with a solution based on naphthol AS-BI and fast red violet LB. The cells were photographed using a Leica DM1000 (Leica Microsystems, Wetzlar, Germany) featuring Leica LAS X software (Leica Microsystems).

### 4.12. Trilineage Differentiation Assay

A trilineage differentiation assay was performed with a STEMdiff Trilineage Differentiation Kit (Stem Cell Technologies, Vancouver, BC, CA), following the manufacturer’s instructions. IPSCs were plated onto Matrigel and the appropriate trilineage medium was added for 5 days to the wells in order to perform endoderm or mesoderm differentiation or for 7 days to induce the ectoderm differentiation. Cells were fixed, stained, and imaged to document their positivity to anti-SOX17 (1:3200, rabbit), anti-NCAM (1:400, rabbit), and anti-BRACHYURY (1:1600, rabbit) (Cell Signaling).

### 4.13. Genome Integrity Assay

Molecular karyotyping of isogenic iPSCs was performed using the hiPSC Genetic Analysis Kit (Stem Cell Technologies ) following the manufacturer’s instructions, in order to detect the most common karyotype abnormalities reported in human iPSCs (Chr 1q, Chr 4p, Chr 8q, Chr 10p, Chr 12p, Chr 17q, Chr 18q, Chr 20q, Chr Xp). DNA was extracted from iPSCs with the QIAamp DNA Blood Mini kit (Qiagen, Hilden, Germany) and quantified with NanoDrop 2000/2000c Spectrophotometers (ThermoFisher). Data were analyzed with a Genetic Analysis Application supplier (Stem Cell Technologies).

### 4.14. TUNEL Assay

The nuclear DNA fragmentation measurement was carried out with a DeadEnd Fluorometric TUNEL System Kit (Promega) following the manufacturer’s instructions. Briefly, iPSCs were fixed with 4% paraformaldehyde for 10 min at room temperature and permeabilized with 0.2% Triton X-100 (Sigma Aldrich) for 10 min. Cells were incubated with Equilibration Buffer, Nucleotide Mix, and rTdT Enzyme at 37 °C for 1 h in order to label DNA strand breaks with fluorescein-12-dUTPAdd. Nuclei were stained with Hoechst 33342 dye (Invitrogen).

### 4.15. MTT Assay

Thiazolyl blue tetrazolium (MTT) (Sigma Aldrich) was used as a colorimetric indicator as MTT is reduced to formazan (a violet-blue water-insoluble molecule) by metabolically active cells. The cells were seeded in 96-well cluster plates and the following day the medium containing MTT powder (5 mg/mL) was added. The cells were then incubated for 2 h and 30 min at 37 °C. After incubation, formazan crystals appeared at the cell surface and were quantified by an EnSpire Multimode Plate Reader (Perkin Elmer, Boston, MA, USA).

### 4.16. Statistical Analyses

Multiple technical replicates and biological replicates were utilized for all experiments and three independent experiments were performed for each assay. Data were represented using mean and standard error (mean ± SEM), and significance was tested using ANOVA (parametric tests) for normally-distributed data and Kruskal–Wallis (nonparametric tests) when normal distribution could not be assessed. GraphPad-Prism software (Prism 8.0.2, GraphPad Software) was used to analyze the data.

## Data Availability

The data that support the findings of this study are available on request. The generated lines should be requested to CC and MT.

## Supplementary Materials

The supporting information can be downloaded at: https://www.mdpi.com/article/10.3390/ijms24097988/s1**.**
