# Supplementary material for: CRISPR/Cas9 and piggyBac Transposon-Based Conversion of a Pathogenic Biallelic TBCD Variant in a Patient-Derived iPSC Line Allows Correction of PEBAT-Related Endophenotypes
Source: Int J Mol Sci. 2023 Apr 28;24(9):7988. doi: 10.3390/ijms24097988 (PMC10178052; doi:10.3390/ijms24097988)
Supplement: Supplementary file 1 [file ijms-24-07988-s001.zip › ijms-2298865-supplementary.pdf]

SUPPLEMENTARY INFORMATION

Supplementary Figure S1: MTT assay and Trilineage differentiation assay **Supplementary Table S1:** List of primers used in this study

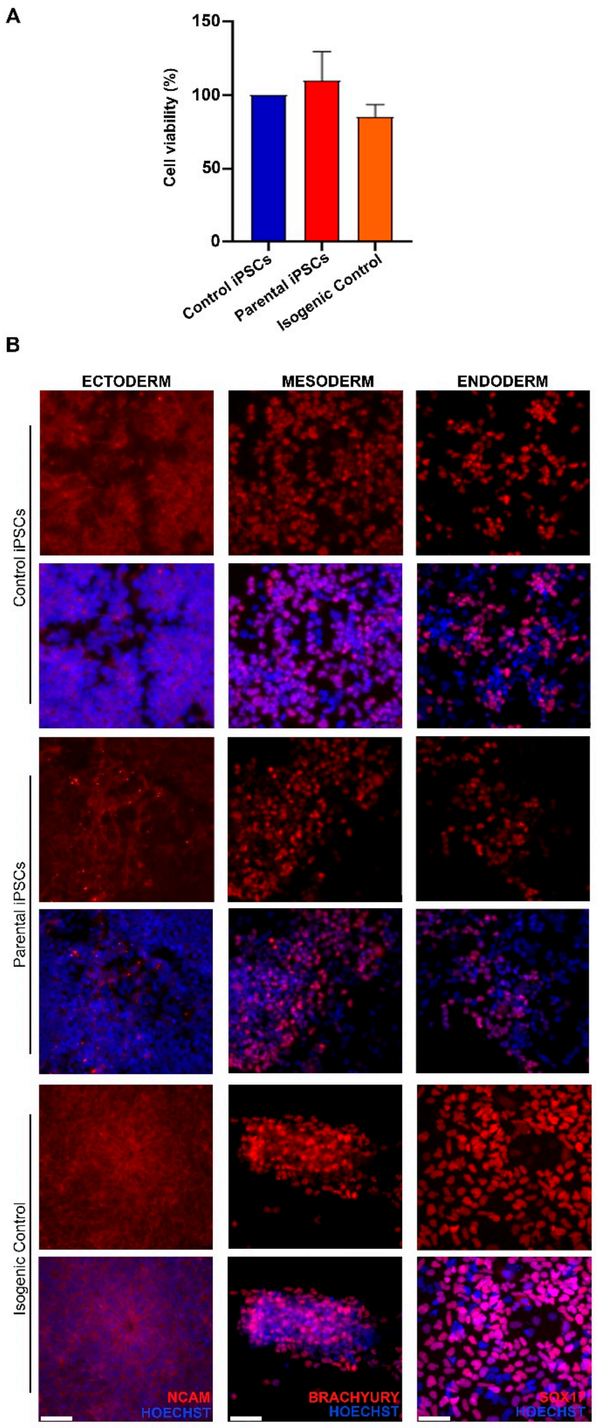

Figure S1: Isogenic iPSC line maintains its proliferative potential and its ability to differentiate into the three embryonic germ layers. (A) Cell proliferation was estimated via MTT assay. The

histogram represents the percentage of viable parental and isogenic iPSCs compared with control cells (control iPSCs, 100%). Data are presented as mean ± SEM (normalized to control), n = 3. Ordinary one-way ANOVA parametric test is used to assess statistical significance. (B) Confocal micrographs of endoderm (SOX17), mesoderm (BRACHYURY) and ectoderm (NCAM) markers show the functional ability of control iPSCs, parental iPSCs and isogenic lines to differentiate into cells belonging to the three germ layers. Scale bar = 50 µm.

Table S1: List of primers used in this study

Primer	Sequence (5'-3')	Applications
P1	ATGTTCAAAGCAGTCGCAATGG	Amplification Genomic DNA of TBCD
P2	TCTCACCTGCTCTCTATTAACC	
TBCDwt	F:TCTGCCACCGTTTCCCGCTGGTGAGTGCCTGC	Mutagenesis
	R: GCAGGCACTCACCAGCGGGAAACGGTGGCAGA	
PUΔTK	F:AACCCTAGAAAGATAGTCTGC	Amplification selection cassette
	R:AACCCTAGAAAGATAATCATA	
P3	AGAAGCTCCTGTCAGGCATCGC	Screening targeted clones
P4	ACAAGGGTAGCGGCGAAGATCC	
P5	AGCAATGTCTCGTACACCTGG	
M-PCR-TBCD	F:CATGTGCTGCTCCCGGCAG	multiplex PCR
	R:AACACAACAGTGCCAGCGAAG	
M-PCR-GAPDH	F:CTACACTGAGCACCAGGTG	multiplex PCR
	R:CCAGCAAGAGCACAAGAGG	
crRNA 1	CCGTTTCCCGCTGGTGAGTGCCT	gRNA (1)
crRNA 2	GCTCACGTGTGTTTGCCGTGTGG	gRNA (2)
OT2	F:AGGATCGTGGATCAGCCTGC	Amplification off-target
	R: ACCTTCTCCCACCTTCCGTG	
OT3	F:GGATTGGGTGCTGTTAAGGGA	Amplification off-target
	R:TCACAAAACACCAGCATCAGA	
OT4	F:ATGCCCTGGTTGCTCCCAGAG	Amplification off-target
	R:CAGGTTTCTGGCCAGAAGAACC	
OT5	F:CTGTCTGACCTGACAAGCCA	

	R:CCGAAATCAGCTTTGGTCTTG	Amplification off-target
OT6	F:TCCCTCCTCCTCCTTTCACTGC	Amplification off-target
	R:CAGCGAGGATCGTGCTGGTTTC	
OT7	F:CTTGCTAGCCACCCACCGAAT	Amplification off-target
	R:TCATCCCTGACCCTAAGCACAAA	
OT8	F:TAACCGCGGGCTACACAGT	Amplification off-target
	R:GCTCTGTAACCTGGGAGAC	
OT9	F:TTCAGTTCGCTGTGCTCGGC	Amplification off-target
	R:CCATCACTAGAGTCCGCTGTG	
OT10	F:CTGGGCCCCTCCACACACAG	Amplification off-target
	R:GGGACAGCTGTTGCAGCTCAGC	
GAPDH	F:TGCACCACCAACTGCTTAGC	qPCR
	R:GGCATGGACTGTGGTCATGAG	
OCT4	F:AGCGAACCAGTATCGAGAAC	qPCR
	R:TTACAGAACCACACTCGGAC	
SOX2	F:AGCTACAGCATGATGCAGGA	qPCR
	R:GGTCATGGAGTTGTACTGCA	
